# Cardiovascular profile and oral submucous fibrosis among long-term tobacco chewers

**DOI:** 10.6026/973206300211201

**Published:** 2025-05-31

**Authors:** Navdeep Singh Kathuria, Gagandeep Kaur Sidhu, Navneet Singh Kathuria, Amandeep Kaur, Parth Sudan, Parveen Sharma

**Affiliations:** 1 Department of General Medicine, Dr. S.S. Tantia Medical College, Hospital and Research Center, Sri Ganganagar, Rajasthan, India; 2 Department of Oral Pathology and Microbiology, Maharaja Ganga Singh Dental College and Research Centre, Sri Ganganagar, Rajasthan, India; 3 Department of Dentistry, Dr. S.S. Tantia Medical College, Hospital and Research Center, Sri Ganganagar, Rajasthan, India; 4 Department of Dentistry, Surendera Dental College and Research Institute, Sri Ganganagar, Rajasthan, India; 5 Departmetnt of Anatomy, Dr. S.S. Tantia Medical College, Hospital and Research Center, Sri Ganganagar, Rajasthan, India

**Keywords:** Electrocardiograph, sinus bradycardia, smokeless tobacco

## Abstract

The cardiovascular profile and incidence of oral submucous fibrosis among 296 individuals with a history of tobacco chewing exceeding 15 years is of interest. Participants underwent comprehensive examinations, including HbA1c, lipid profile, ECG and oral assessments. Sinus bradycardia was observed in 16% of cases, with a significant correlation to the duration of tobacco use. Oral submucous fibrosis was found in 22.29% of subjects, alongside other notable oral lesions. The findings highlight the substantial cardiovascular and oral health risks associated with prolonged smokeless tobacco consumption.

## Background:

India, a nation of significant population, ranks second globally in terms of population size. Additionally, it holds the position of being the third-largest tobacco producer as well as consumer worldwide. Considering the serious health risks associated with tobacco use, it is imperative to understand the causes of tobacco use and create policies targeted to mitigate its prevalence. The interconnection of factors is particularly significant in the context of developing nations [1]. Similarly in India, where the usage of tobacco remains high despite the acknowledgment of its detrimental effects [1]. On the basis of previous small-scale research, the World Health Organisation estimated that 65% of males and 33% of women used some sort of tobacco products such as cigarettes, bidis, waterpipe (hookah or shisha), chewing tobacco (khaini or gutka) or snuff (powdered tobacco) [2]. A survey named global tobacco survey (GATS) was carried out in India in year 2009-2010 reported that smokeless tobacco (SLT) in smokeless form was most commonly used revealing its prevalence being 26% (33% men;18%women) and 21% use it routinely [3]. It is clear that tobacco usage in India has remained substantially unchanged despite several tobacco prevention and control strategies implemented over the last 50 years. Furthermore, relatively more prevalence is found in rural in comparison to urban part of country. However, usage in tribal area out-numbers to prevalence of usage in urban and rural counterparts [4]. Moreover, the field of literature provides extensive evidence about the detrimental consequences of tobacco smoking. Cigarette smoke, consisting of over 4000 chemical compounds, has been shown to significantly impair cardiovascular function. Conversely, there is a scarcity of published data regarding the adverse effects of smokeless tobacco [5]. Therefore, it is of interest to evaluate the cardiovascular profile and assess the incidence of oral submucous fibrosis amongst individuals with a history of tobacco chewing for more than 15 years, in order to understand the potential long-term health consequences of prolonged tobacco chewing.

## Materials and Methods:

The current observational research was conducted on a sample of 296 individuals who had history of tobacco chewing for more than 15 years and reported at tertiary Medical College and Research centre. Before enrollment, an informed consent was obtained from the subjects. Inclusion criteria comprised of patients giving informed consents and all patients who had history of tobacco chewing for more than 15years. Exclusion criteria comprised of patient with history of active smoking or having any other addiction disorder and patients with any comorbidities. The research had a cohort of 350 participants, of which 296 people granted their permission to participate. The ethical clearance (Reference number: LNMC&RC/dean/2019/ethics/109) was obtained from the institutional ethical committee before commencement of the present study. The research included patients who met the specified inclusion criteria and a comprehensive case history and clinical examination were conducted. The subjects underwent clinical examination and laboratory investigations that included fasting/random blood sugar (F/RBS), renal function test (RFT) and HbA1c profile, lipid profile, ECG. Relevant clinical data including oral manifestations were recorded in a structured proforma. The smokeless forms of tobacco include many products such as gutkha, betel quid, kharra, khaini, pan masala and similar alternatives. Native to some parts of Maharashtra and Central India, kharra is a tobacco mixture made of processed tobacco, lime and either betel nut or areca nut. The material is frequently swallowed whole and frequently chewed. Betel leaf, areca nut, slaked lime, tobacco, catechu and additional spices-which are chosen according to personal preferences-combine to form betel quid [6]. Khaini is a tobacco-based product that comprises flakes of tobacco in roasted form which are combined with slaked lime. The produced combination is stored inside the lower part of buccal or labial sulcus. A commercially produced moist smokeless tobacco product (MSTP) called "gutkha" is made up of a mixture of tobacco, areca nut and other sauces. It comes in a variety of tastes and is frequently presented in eye-catching pouches. Another one is "pan masala” that is a composite blend of betel quid, including areca nut and several seasonings, with the potential inclusion of tobacco. The concoction is orally consumed by chewing and suctions [7]. The participants' self-reporting provided the information on tobacco use. The participants were also required to self-report their monthly income. The principal financial provider or the head of the household's income was documented if the individual was unemployed. The proforma was provided in both English and Hindi languages, including inquiries pertaining to the socio-demographic characteristics as well as the use patterns of tobacco products. The data was inputted into a spreadsheet with the software Microsoft Excel. The Chi-square test was used to analyze the pertinent data, with a significance level of p < 0.05.

## Results:

In the present study, among the enrolled tobacco chewers for more than 15 years, 4 patients (1.33%) were aged <30 years, 55 (18.7%) were aged 31-40 years, 84 (28.34%) were aged 41-50 years, 84 (28.34%) were aged 51-60 years, 54 (18.21%) were aged 61-70 and 15 (5.06%) patients were above 70 years of age (Table 1). The mean value of lipid profile total, TG, HDL and LDL in the present study was 139.86±23.51, 96.35±18.09, 45.88 ± 6.05 and 87.55 ± 15.42 respectively. The mean values of F/RBS, Urea, Creatinine and HbA1c in the present study were 102.15 ± 9.30, 29.69±14.05 and 0.99 ± 0.129 and 5.36 ± 0.482 respectively. All the values were within normal limits (Table 2). The ECG findings in the present study revealed sinus bradycardia in 48 (16%), other findings in 31 (10.3%) cases and in 221 (73.7%) cases, reports were within normal limits (WNL) (Table 3). Among total 296 study subjects, 125 (43%) were gutkha chewers, out of which 21 subjects were found to have sinus bradycardia on ECG; 126 (42%) were khaini chewers, out of which 20 subjects were found to have sinus bradycardia on ECG and remaining 45 (15%) subjects were in habit of chewing Zarda, among whom 6 subjects were diagnosed with sinus bradycardia, however the comparison was found to be statistically not significant (Table 4). The present found a significant correlation between time duration of tobacco chewing and sinus bradycardia and other ECG findings (p<0.01) (Table 5). On oral examination, oral submucous fibrosis was found in 66 (22.29%) of study subjects, tongue discoloration was found in 116 (39.18%) of study subjects, palate discoloration was in 77 (26%), high arch palate was seen in 5 (1.68%) cases, oral ulcers were in 16 (5.4%) cases and other lesions (leukoplakia, erythroplakia, tobacco pouch keratosis, nicotine stomatitis, smoker's palate) were seen in 21 (7.09%) cases. The present found that study found a significant correlation between time duration of tobacco chewing and oral submucous fibrosis (p<0.01) (Table 6).

## Discussion:

Tobacco consumption is a recognized risk factor that is strongly linked to the development of non-communicable diseases (NCDs) that include diabetes mellitus, hypertension and various types of cancer (oral squamous cell carcinoma, esophageal, pharyngeal, laryngeal and pancreatic and stomach cancer), chronic respiratory disease, cardiovascular disease and asthma. Although smoking cigarettes is still the most frequent manner that youth worldwide use tobacco products, however, using other tobacco products is also prevalent [8, 9]. In contemporary times, the introduction of industrialization have a considerable impact on the lifestyle patterns of individuals, thereby leading to the emergence of non-communicable illnesses among younger demographics. While non-modifiable features are beyond intervention, it is essential to provide immediate and substantial attention to modifiable attributes in order to mitigate the impact of these insidious threats. The contemporary period is seeing a transformation in lifestyle patterns, which is leading the younger generation to engage in riskier behaviors that were traditionally associated with older individuals and their associated health issues [10]. Thus, in reference to it, the present prospective observational study was commenced to appraise the clinical profile of tobacco chewers who were consuming it for more than 15 years. In the present study, tobacco chewers with over 15 years of habit were predominantly found in two age groups: 41-50 years and 51-60 years, each accounting for 28.34% of the total, with 84 patients in each group. The next most common age groups were 31-40 years, making up 18.7% of the total with 55 patients and 61-70 years, comprising 18.21% of the total with 54 patients (Table 1). As 41-60 years age group males forms the major part of bread-earning population in our country, various factors such as the nature of work, hours of work put this age group under stress and anxiety. Moreover, there are certain misconceptions pertaining to tobacco use, such as the belief that smoking alleviates anxiety and stress, creates pleasurable sensations, promotes skeletal muscle relaxation and enhances attention and so forth [11]. The demographic characterized by their means of earning a living has the highest prevalence of tobacco use, mostly driven by the perceived benefits attributed to its usage [11, 12]. In a previous research done by Mitra *et al.* [13] participants reported average tobacco consumption duration of 5 to 6 years. In contrast, the subjects involved in the current study reported a tobacco chewing habit spanning over 15 years. Therefore, the present study is unique as it one and only to evaluate the electrocardiographic and biochemical profile of tobacco chewers who are consuming it over a long period of 15 years. There exists a correlation of cigarette smoking with chronic kidney disease (CKD) in relation to cardiovascular morbidity and the progression of atherosclerosis [14]. The mean value of lipid profile i.e., total cholesterol, triglycerides (TG), LDL and HDL in the present study was 139.86 ± 23.51, 96.35 ± 18.09, 87.55 ± 15.42 and 45.88±6.05and mg/dl respectively which were within normal limits. In contrast, Brischetto *et al.* [15] proposed a mechanism explaining the changes in lipid profile associated with cigarette use. Nicotine induces the secretion of adrenaline from the adrenal cortex, thereby enhancing the activation of adenyl cyclase in adipose tissue. Consequently, this leads to the breakdown of triglycerides (TG) held in adipose tissue and the subsequent liberation of Free Fatty Acids (FFA's). Plasma albumin exhibits binds affinity towards the liberated free fatty acids (FFAs), facilitating their transportation to different bodily regions, particularly the liver. This process triggers hepatic synthesis, leading to the secretion of cholesterol and very low-density lipoprotein (VLDL), thus resulting in elevated levels of triglycerides (TG). The observed drop in plasma high-density lipoprotein (HDL) levels, along with an increase in plasma triglycerides (TG) and very low-density lipoprotein (VLDL), may be attributable to an elevation in the concentration of plasma free fatty acids (FFAs) [15]. In Waingade *et al.*'s study, [16] they analyzed and contrasted the effects of tobacco chewing on the lipid profiles of those who chew tobacco with a control group. In line with findings from earlier research on blood samples, the lipid components (total cholesterol, triglycerides, high-density lipoprotein, low-density lipoprotein and very low-density lipoprotein) showed higher mean values in tobacco chewers compared to the control group. However, present study did not find this association which could be due to exclusion of patients with any comorbidity. In the current investigation, the electrocardiogram (ECG) results revealed that sinus bradycardia and WNL were seen in 11.15%, 16.21% and 72.64% of the participants, respectively. The observed alterations in heart rate, QRS length and TP interval align with the findings reported by Venkatesh *et al.* [17] who conducted a comparative research on the electrocardiogram (ECG) variations between smokers and those without smoking habits. When compared to the control group, smokers exhibited a statistically significant decrease in the T-P interval and a statistically significant elevation in heart rate. The statistical analysis showed that the P-wave, P-R interval, QTC interval, QRS frontal axis, ST segment and T-wave did not exhibit any significant findings. The research conducted by Siddiqui *et al.* [18] reveals that those who smoke or chew tobacco have a notable rise in heart rate, along with a substantial reduction in the TP interval and length of the QRS complex, as compared to the control group. No significant changes were seen in other parameters, including P wave amplitude (in millivolts), P wave length (in seconds), QT interval (in seconds), T wave amplitude (in millivolts) and ST wave amplitude (in millivolts), when analyzed using an unpaired t-test. The findings of the current study on the elevation of heart rate in smokers are consistent with the research conducted by Moliterno *et al.* [19] In recent decades, extensive research has resulted in the discovery of several risk factors for cardiovascular disease that are linked to both cigarette smoking and its biomarkers. The relationship between the use of smokeless tobacco (ST) and other developing tobacco products and the risk of cardiovascular disease, as well as the biomarkers linked with this risk, lack clarity in current academic literature. The stiffness of arterial walls serves as a significant early indicator for the detrimental impact of DT on cardiovascular morbidity. Pulse wave analysis (PWA) is a non-invasive technique used for the assessment of arterial stiffness. The primary discovery in this investigation indicates that the process of steeping tobacco overnight was correlated with a significant reduction in heart rate [20]. The current investigation discovered comparable results for sinus bradycardia. The decrease in heart rate seen in this research, as shown by the electrocardiogram (ECG) results, may be attributed to the cessation of nightly use of smokeless tobacco. It is worth noting that bradycardia, a recognised symptom of nicotine withdrawal, might potentially explain this finding. Both smoking and smokeless tobacco have been shown to cause an elevation in heart rate, which may be attributed to either the local release of norepinephrine from adrenergic axon terminals inside the heart or an enhanced release of catecholamine. The requirement for a definitive determination is imperative due to the existence of conflicting data reported in various investigations [21, 22]. On oral examination, submucous fibrosis was found in 22.29% of study subjects, tongue discoloration was found in 39.18% of study subjects, palate discoloration was in 26%, high arch palate was seen in 1.68% cases, oral ulcers were in 5.4% cases and other lesions were seen in 7.09% cases. In this study, participants who were native to Central India, most of them consumed kharra [6] which is a tobacco mixture made of processed tobacco, lime and either betel nut or areca nut. The present found that a significant correlation between time duration of tobacco chewing that contained betel nut or areca nut and oral submucous fibrosis. Similarly, various studies conducted by Jha *et al.* [23] Pandya *et al.* [24] Hosein *et al.* [25] Malvania *et al.* [26] revealed that severity of OSMF increases as duration of tobacco consumption. Study also found that initiating age of tobacco chewing is early adolescent despite of law inhibiting them. This group should be the target age group for inhibition campaigns and awareness should be created among the early age group of 12 years and above i.e., among teenagers. Tobacco has been used by humanity in various manifestations for many generations. The use of this entity often starts during the early stages of human development. In recent years, there has been an upward trajectory in the prevalence of tobacco use, particularly in smokeless forms, within the Indian context. The precise prevalence of tobacco use among teenagers in India remains unknown at a national level, despite the existence of many surveys conducted in different regions of the country. There exists a need to cultivate awareness among the younger generation about the detrimental health consequences associated with the use of tobacco products.

## Conclusion:

Smokeless tobacco use has significant cardiovascular effects, such as sinus bradycardia, indicating serious health risks. Given the high prevalence of tobacco use in developing countries like India, there is an urgent need for targeted strategies to reduce its widespread use and associated health consequences.

## Figures and Tables

**Table 1:** Age of the participants

**Age Group (in years)**	**N**	**%**
<30	4	1.33
31-40	55	18.7
41-50	84	28.34
51-60	84	28.34
61-70	54	18.21
>70	15	5.06
Total	296	100

**Table 2:** Laboratory investigations

**Laboratory investigations**				
**Variables**	**Minimum**	**Maximum**	**Mean**	**Std. Deviation**
Total Cholesterol (TC) mg/dl	110	180	139.86	23.515
Triglyceride (TG)	59	149	96.35	18.091
High-density lipoprotein (HDL)	25	52	45.88	6.052
Low-density lipoprotein (LDL)	57	123	87.55	15.423
Fasting/Random Blood Sugar mg/dl	76	135	102.15	9.306
Urea mg/dl	15	89	29.69	14.054
Creatinine mg/dl	0	2	0.99	0.129
HBA1c%	5	6	5.36	0.482

**Table 3:** ECG findings among the study subjects

**ECG Findings**	**Number**	**Percentage**
Others	31	10.3
Sinus Bradycardia	48	16
Within normal limit (WNL)	221	73.7

**Table 4:** Comparison of type of tobacco according to ECG findings

**Type**		**ECG Finding**			**Total**
		**Others**	**Sinus Bradycardia**	**WNL**	
Gutkha	N	14	21	90	125
Khaini	N	12	20	94	126
Zarda	N	6	6	33	45
Total	N	32	47	217	296
Chi Square	0.79				
p value	0.94				

**Table 5:** Association of duration of tobacco chewing with ECG findings

**Duration**		**Others**	**Sinus Bradycardia**	**WNL**	**Total**
	N	3	0	85	88
15-20	%	9.40%	0.00%	39.20%	29.70%
	N	4	0	64	68
21-25	%	12.50%	0.00%	29.50%	23.00%
	N	11	16	43	70
26-30	%	34.40%	34.00%	19.80%	23.60%
	N	5	12	22	39
31-35	%	15.60%	25.50%	10.10%	13.20%
	N	7	12	3	22
36-40	%	21.90%	25.50%	1.40%	7.40%
	N	1	3	0	4
41-45	%	3.10%	6.40%	0.00%	1.40%
	N	1	4	0	5
>45	%	3.10%	8.50%	0.00%	1.70%
	N	32	47	217	296
Total	%	100.00%	100.00%	100.00%	100.00%
Chi Square	123.31				
p value	<0.01				

**Table 6:** Association of duration of tobacco chewing with Oral submucous fibrosis (OSMF)

		**OSMF**	
**Duration**		**No**	**Yes**
	N	86	2
15-20	%	37.40%	3.00%
	N	62	6
21-25	%	27.00%	9.10%
	N	48	22
26-30	%	20.90%	33.30%
	N	21	18
31-35	%	9.10%	27.30%
	N	9	13
36-40	%	3.90%	19.70%
	N	3	1
41-45	%	1.30%	1.50%
	N	1	4
>45	%	0.40%	6.10%
	N	230	66
Total	%	100.00%	100.00%
Chi Square		70.49	
p value		<0.01	
